# Epipharyngeal Abrasive Therapy (EAT) Improved Premature Ventricular Contractions (PVCs) and Brain Fog Associated With Long COVID: A Case Report

**DOI:** 10.7759/cureus.102533

**Published:** 2026-01-29

**Authors:** Ito Hirobumi

**Affiliations:** 1 Otolaryngology, Ito ENT Clinic, Funabashi, JPN

**Keywords:** adjustment effect., brain fog, eat field theory, eat reflex adjustment 3-phase model, epipharyngeal abrasive therapy (eat), immediate effect, long covid, time-dependent effect, ventricular premature contractions (pvc)

## Abstract

A 50-year-old man with premature ventricular contractions (PVCs) detected before coronavirus disease 2019 (COVID-19) infection developed persistent fatigue and brain fog following COVID-19, which caused difficulty in continuing his work. Epipharyngeal abrasive therapy (EAT) was started without pharmacological treatment. Serial Holter electrocardiography demonstrated a marked reduction in PVC burden after continued EAT. In contrast, improvement in brain fog and recovery of work motivation occurred at a later stage, eventually allowing him to return to work. PVCs are considered an autonomic-sensitive peripheral manifestation, whereas brain fog likely represents dysfunction of central autonomic networks. The temporal dissociation observed between early improvement in PVCs and delayed cognitive recovery indicates that EAT may exert time-dependent effects on peripheral and central autonomic regulation. This case suggests that EAT could serve as a non-pharmacological and minimally invasive therapeutic option for both peripheral and central symptoms associated with autonomic dysfunction in Long COVID.

## Introduction

Premature ventricular contractions (PVCs) are common arrhythmias that occur in individuals ranging from healthy subjects to patients with underlying structural heart disease, and they may cause symptoms such as palpitations and chest discomfort. Treatment strategies for PVCs include observation, pharmacological therapy, or catheter ablation, depending on symptom severity, frequency, and the presence of underlying cardiac conditions. However, there are cases in which pharmacological therapy is unnecessary or ineffective, or when patients prefer to avoid invasive treatments [1].

In recent years, persistent and diverse symptoms following coronavirus disease 2019 (COVID-19) infection, collectively referred to as post-COVID-19 condition (Long COVID), have received considerable attention. Symptoms such as fatigue, palpitations, dyspnea, and orthostatic intolerance have been linked to autonomic dysfunction, and associations with the exacerbation or new onset of arrhythmias have also been reported [2]. Nevertheless, the relationship between Long COVID and PVCs, as well as the optimal treatment strategies, remains poorly understood.

Epipharyngeal abrasive therapy (EAT) has been widely used in otolaryngology for the treatment of chronic nasopharyngitis. Recent studies have suggested that EAT may influence autonomic function and systemic inflammation, and it has gained attention as a potential adjunctive therapy for Long COVID [3]. However, reports describing the improvement of arrhythmias, particularly PVCs, following EAT, remain extremely limited. This report describes a case in which continued EAT, initiated for Long COVID-related fatigue in a patient with pre-existing PVCs, resulted in marked improvement of PVCs without pharmacological therapy, followed by delayed improvement of brain fog. The report provides clinically meaningful insights into the staged effects of EAT.

## Case presentation

A 50-year-old man underwent Holter electrocardiography in November 2023, which revealed frequent PVCs. Further electrocardiography and echocardiography showed no structural heart disease or cardiac dysfunction other than the PVCs. Laboratory testing revealed no significant abnormalities. At that time, the patient was informed that catheter ablation might be considered if PVCs did not improve. In April 2024, the patient contracted COVID-19 and subsequently developed persistent fatigue, exhaustion, difficulty concentrating, and brain fog. These symptoms persisted, leading to marked impairment of daily activities and work, with substantial deterioration in quality of life.

In June 2024, the patient visited the author’s clinic and began EAT. EAT was performed using a standardized protocol commonly applied in clinical practice. Each session involved gentle mechanical abrasion of the epipharyngeal mucosa using a cotton swab soaked in a 1% zinc chloride solution, with an approximate duration of 5-10 minutes per session. Treatments were administered once or twice weekly, depending on symptom severity and patient tolerance. No procedural complications were observed during the treatment course. Initial endoscopic examination revealed mild findings of chronic nasopharingitis, with minimal bleeding during abrasion.

**Figure 1:**
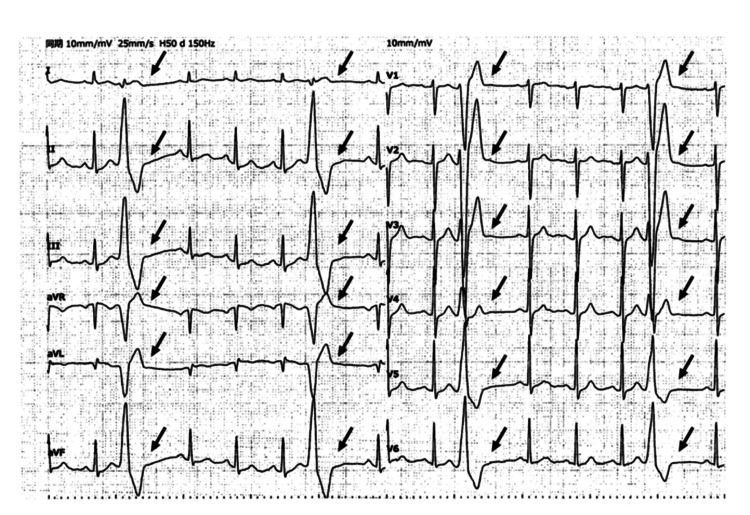
12-lead ECG after EAT treatment This 12-lead ECG was performed after the second Holter monitoring session following EAT treatment. While PVCs are visible at the arrow locations, sinus rhythm predominates compared to pre-EAT treatment, and PVCs are markedly reduced. No significant ST elevation or ST depression suggestive of acute ischemia is observed. This ECG visually demonstrates the post-treatment reduction in PVC morphology and occurrence frequency, corresponding to the decreased PVC rate confirmed by the Holter ECG ECG: electrocardiogram; EAT: epipharyngeal abrasive therapy; PVC: premature ventricular contraction Image credit: author

EAT was performed once or twice weekly. Between June and November 2024, a total of 26 EAT sessions were administered. A second Holter electrocardiogram performed in November 2024 demonstrated marked improvement in PVCs. The PVC burden decreased from 14.3% to 3.3%, representing a 68.8% reduction. At that time, laboratory testing, including complete blood count, liver and renal function tests, metabolic parameters, and cardiac biomarkers, revealed no clinically significant abnormalities (Table 1). No pharmacological treatment for PVCs was administered throughout the clinical course (Figure 1, Table 2).

**Table 1:** Laboratory findings at Holter ECG evaluation following EAT treatment This table summarizes the results of laboratory tests performed during Holter ECG evaluation following EAT treatment. Items include complete blood count, liver and kidney function tests, metabolic markers, electrolyte levels, glucose indicators, lipid profile, and cardiac biomarkers. Reference ranges corresponding to all values are provided to facilitate clinical interpretation. Overall, no clinically significant abnormalities were observed. Reference ranges are based on our facility's standards at the time of testing ECG: electrocardiogram; EAT: epipharyngeal abrasive therapy; AST: aspartate aminotransferase; GOT: glutamic-oxaloacetic transaminase; ALT: alanine aminotransferase; GPT: glutamic-pyruvic transaminase; ALP: alkaline phosphatase; HDL: high-density lipoprotein; LDL: low-density lipoprotein; eGFR: estimated glomerular filtration rate; HbA1c: hemoglobin A1C; WBC: white blood cells; RBC:P red blood cells; MCV: mean corpuscular volume; MCH: mean corpuscular hemoglobin; MCHC: mean corpuscular hemoglobin concentration; NT-proBNP: N-terminal pro-B-type natriuretic peptide

Parameter	Reference range	Unit	Value	Interpretation
AST (GOT)	11–35	U/L	30	Within normal range
ALT (GPT)	6–39	U/L	48	Mildly elevated
ALP	38–113	U/L	55	Normal
γ-GTP	≤73	U/L	39	Normal
Triglycerides	30–149	mg/dL	88	Normal
HDL cholesterol	40–79	mg/dL	54	Normal
LDL cholesterol	70–139	mg/dL	134	Upper-normal range
Uric acid	3.6–7.0	mg/dL	5.9	Normal
Creatinine	0.61–1.04	mg/dL	0.88	Normal renal function
eGFR (CRE)	≥60	mL/min/1.73m²	72.6	Preserved renal function
Potassium	3.5–5.0	mEq/L	3.8	Normal
HbA1c	4.6–6.2	%	5.4	Normal glycemic control
WBC	3900–9700	/µL	3600	Slightly below reference range
RBC	438–577	×10⁴/µL	505	Normal
Hemoglobin	13.6–18.3	g/dL	15.6	Normal
Hematocrit	40.4–51.9	%	49.1	Normal
MCV	83–101	fL	97	Normocytic
MCH	28.2–34.7	pg	30.9	Normal
MCHC	31.8–36.4	g/dL	31.8	Lower-normal limit
Platelet count	14.0–37.9	×10⁴/µL	16.3	Normal
NT-proBNP	≤125	pg/mL	13	No evidence of heart failure

**Table 2:** Comparison of 24-hour Holter ECG findings before and after EAT ^*^Reference values indicate commonly cited ranges or thresholds used for clinical interpretation rather than strict “normal limits,” as many Holter parameters depend on age, activity level, and clinical context. ^†^Total heartbeats and maximum heart rate vary with physical activity and recording duration and therefore do not have fixed normal ranges. ^‡^Short sinus pauses (<3 s) may be observed in healthy individuals, particularly during sleep; longer pauses are generally considered pathological depending on symptoms. This table presents a side-by-side comparison of heart rate indices and arrhythmic findings obtained from 24-hour ambulatory electrocardiography performed before and after EAT. Changes are interpreted in relation to overall heart rate dynamics and arrhythmic burden rather than isolated absolute values. The table highlights a substantial reduction in PVCs following EAT, together with preserved rhythm stability and absence of newly emergent atrial arrhythmias or clinically significant conduction abnormalities. Laboratory testing revealed no significant abnormalities ECG: electrocardiogram; EAT: epipharyngeal abrasive therapy; PVCs: premature ventricular contractions; PVC burden: percentage of PVCs relative to total heartbeats during the recording period; PACs: premature atrial contractions - atrial ectopic beats originating outside the sinoatrial node; AF/AF: atrial fibrillation/flutter: supraventricular arrhythmias characterized by disorganized (AF) or macroreentrant (AFL) atrial activity; RR prolongation (sinus pause): transient prolongation of the RR interval due to delayed sinus node activity; ST changes: deviations of the ST segment from baseline observed during ambulatory ECG monitoring

Parameter	Reference/typical values^*^	Before EAT	After EAT	Interpretation
Effective recording time	Standard short-term ambulatory ECG: ~24 h	23:41:12	23:59:57	Adequate and comparable recordings
Total heartbeats	No fixed normal range^†^	98,097	96,444	Slight reduction, consistent with lower mean HR
Mean heart rate (HR)	Resting adult HR: 60-100 bpm	69 bpm	68 bpm	Stable, mildly decreased
Maximum heart rate	Activity-dependent; no fixed normal^†^	110 bpm	103 bpm	Reduced peak HR, suggesting decreased sympathetic drive
Minimum heart rate	Normal nocturnal HR: ~40-60 bpm	49 bpm	46 bpm	Within the physiological nocturnal range
PVC count (% burden)	PVCs may be present in healthy subjects; clinical concern often occurs at ≥10% burden	14,072 (14.3%)	3,219 (3.3%)	Marked reduction to below the high-burden range
PAC count	No uniform cut-off; “frequent PACs” variably defined	8	8	Unchanged; very low frequency
AF/AFL	None	None	None	No atrial tachyarrhythmia observed
RR prolongation (pause)	Sinus pause >3 s is often considered abnormal^‡^	1 episode	Not observed	Disappearance of the transient pause
ST changes	ST analysis on Holter is context-dependent	Mild fluctuations	No ischemic changes	No evidence of myocardial ischemia

In contrast, subjective symptoms of fatigue showed little improvement during the first four months of EAT. Thereafter, following the improvement in PVCs, the patient gradually experienced alleviation of brain fog and a recovery of daytime activity and motivation. Approximately one year after starting EAT, these central symptoms continued to improve, and by June 2025, the patient began considering a return to work. As of September 2025, a total of 77 EAT sessions had been completed, and the patient ultimately returned to work.

## Discussion

In this report, a patient with PVCs identified before COVID-19 infection developed persistent fatigue and brain fog following COVID-19. The primary observations of this case include an objective reduction in PVC burden documented through serial Holter electrocardiography and a delayed, subjective improvement in brain fog and functional recovery. The interpretations regarding autonomic regulation and central network involvement described below are theoretical in nature and aim to provide a conceptual framework rather than establish causality. Continued EAT resulted in objective improvement of PVCs without pharmacological therapy. Importantly, improvement in brain fog and work motivation occurred after the improvement in PVCs, representing a key clinical feature of this case.

Although strict normal ranges are not defined for many Holter parameters, the PVC burden in this case decreased from a clinically significant high-burden range (>10%) to a low-burden range following EAT, accompanied by stabilization of heart rate dynamics and no emergence of atrial arrhythmias. A high PVC burden is clinically relevant because multiple studies have demonstrated an association between frequent PVCs and the development of left ventricular dysfunction, commonly referred to as PVC-induced cardiomyopathy. Although PVCs can be observed in healthy individuals, prior reports suggest that a burden of approximately 10% or more of total heartbeats represents a threshold above which the risk of ventricular dysfunction increases, with some studies proposing higher cutoffs in the range of 16-24% for predicting cardiomyopathy [4,5]. Importantly, reducing the PVC burden below this threshold has been shown to improve or normalize ventricular function, even in the absence of structural heart disease [6]. In our case, the marked reduction in PVC burden from 14.3% to 3.3% following EAT supports the conclusion that the observed improvement was clinically meaningful rather than incidental.

PVCs are known to be strongly influenced by autonomic nervous system balance, particularly in the absence of structural heart disease. Sympathetic predominance enhances myocardial automaticity and excitability, thereby increasing PVC frequency, whereas increased parasympathetic (vagal) influence promotes the electrical stability of the myocardium and suppression of PVCs [7]. The relatively rapid improvement in PVCs observed in this case suggests that autonomic reflex modulation induced by EAT may preferentially influence rapidly responsive peripheral systems such as cardiac electrophysiology. Notably, this improvement is unlikely to reflect a direct cardiac effect of EAT; instead, it reflects recalibration of autonomic reflex pathways mediated through vagal and sympathetic circuits originating in the nasopharyngeal region. Given the absence of structural heart disease, myocardial ischemia, or atrial arrhythmias, the PVCs in this patient were therefore considered autonomic-dependent.

In contrast, Long COVID is frequently associated with fatigue, palpitations, orthostatic intolerance, and brain fog, where autonomic dysfunction, chronic inflammation, and neuroinflammation are implicated. Recent neuroimaging studies suggest that alterations in functional connectivity of central autonomic networks, including the salience network (SN), may be associated with cognitive impairment and reduced motivation in Long COVID [8-11]. The SN, centered on the anterior insula and anterior cingulate cortex, plays a key role in integrating interoceptive and autonomic information and in switching between the default mode network and task-positive networks [8,12,13]. These central symptoms often require longer recovery periods than peripheral symptoms and represent an important contributor to the refractory nature of Long COVID.

Chronic nasopharyngitis has recently been conceptualized not merely as a localized inflammatory condition but as a systemic regulatory disorder involving the autonomic, immune, and endocrine systems. The epipharynx is anatomically rich in afferent fibers from the trigeminal, glossopharyngeal, and vagus nerves, and persistent stimulation arising from this region may influence global homeostatic regulation via the brainstem, hypothalamus, and limbic system. Ito has proposed that sustained afferent input from chronic nasopharyngitis places a continuous allostatic load on autonomic centers, leading to exaggerated reflex responses or reflex fatigue, and has characterized EAT as a reflex-based regulatory therapy [14].

**Figure 2:**
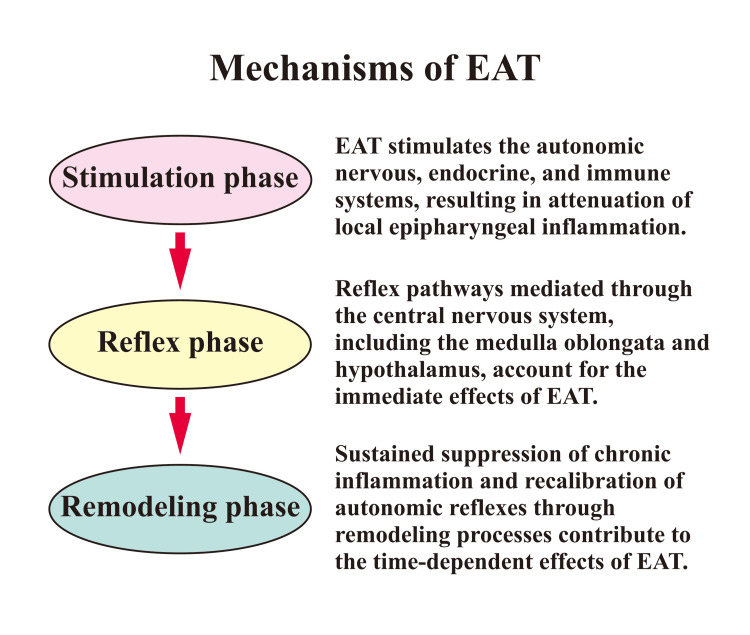
Proposed phased mechanisms of epipharyngeal abrasive therapy (EAT) In the stimulation phase, mechanical and chemical abrasion of the epipharyngeal mucosa generates afferent inputs via Aδ and C fibers to the nucleus tractus solitarius, activating central autonomic regulation. In the reflex phase, autonomic reflexes mediated through the medulla oblongata and hypothalamus produce immediate and time-dependent systemic effects, with reflex patterns varying according to stimulation conditions. In the remodeling phase, continued EAT is associated with attenuation of chronic inflammation and qualitative recalibration of autonomic reflex circuits, contributing to delayed and sustained clinical effects. Immediate effects may be observed concurrently during the stimulation and reflex phases. In contrast, time-dependent effects arising in the reflex phase may become clearly apparent only after progression into the remodeling phase. Accordingly, differences between immediate and time-dependent effects should not be regarded as contradictory but rather as phenomena reflecting phase-dependent temporal dynamics during the therapeutic process. This conceptual figure is based on previously proposed models of epipharyngeal abrasive therapy and autonomic reflex regulation [14-16]. Image credit: author

According to the EAT Reflex Adjustment Three-Phase Model, the effects of EAT can be categorized into three stages: (1) immediate effects, (2) time-dependent effects, and (3) adjustment effects. Immediate and time-dependent effects primarily reflect quantitative alterations in autonomic reflex responsiveness, whereas adjustment effects represent qualitative reorganization of autonomic reflex circuits. This model suggests that EAT induces plastic reconfiguration of regulatory mechanisms rather than simply increasing or decreasing autonomic tone [15]. In the present case, improvement of premature ventricular contractions, an autonomic-dependent peripheral manifestation, preceded improvement in brain fog, which likely reflects progressive recalibration of central neural networks, including the SN (Figure 2). This temporal sequence provides clinical support for the multi-layered effects of EAT unfolding across different time scales. Nonetheless, alternative explanations for the observed clinical improvement should be considered. These include spontaneous recovery following acute COVID-19 infection, regression to the mean in PVC burden, placebo effects, or natural fluctuations of autonomic symptoms over time. Given the single-case design and absence of a control condition, these factors cannot be excluded, and the findings should therefore be interpreted with appropriate caution.

The clinical course of this case is closely consistent with the EAT Reflex Adjustment Three-Phase Model. The early improvement in PVCs can be interpreted as resulting from rapid recalibration of autonomic reflex pathways at the brainstem and peripheral levels, leading to attenuation of sympathetic predominance and stabilization of myocardial excitability. Because PVCs represent autonomic-dependent peripheral manifestations with relatively short reflex arcs, clinical improvement may become apparent during the early phases of reflex adjustment. In contrast, improvement in brain fog and work motivation emerged at a later stage. Within this framework, adjustment effects are thought to develop gradually through neuroplastic reorganization of central regulatory networks, and the delayed improvement of central symptoms observed in this case is therefore physiologically plausible.

From the perspective of the EAT Field Theory, EAT represents a multisystem intervention that simultaneously modulates the autonomic nervous system, immune system (nasopharynx-associated lymphoid tissue, NALT), and endocrine systems (including the hypothalamic-pituitary-adrenal axis, HPA axis, and the sympathetic-adrenal-medullary system, SAM system) through localized intervention at the nasopharynx [16]. Suppression of chronic nasopharyngeal inflammation may reduce persistent inflammatory and neural afferent input to brainstem autonomic nuclei, thereby lowering chronic allostatic load. This reduction in afferent noise may create a physiological environment that facilitates subsequent recalibration and reorganization of central autonomic and cognitive networks, occurring over longer time scales than peripheral reflex adjustments. McEwen’s work demonstrates that chronic stress induces reversible structural remodeling of hippocampal CA3 pyramidal neurons, providing a neuroanatomical substrate through which sustained allostatic load can alter cognition and behavior without irreversible neuronal loss [17].

From a central network perspective, brain fog in Long COVID can be understood as a functional disturbance of the SN, which integrates interoceptive and autonomic inputs and governs the switching between the default mode network (DMN) and task-positive networks (TPN). Persistent inflammation of the nasopharyngeal mucosa may provide continuous afferent input to brainstem autonomic nuclei, including the nucleus tractus solitarius and the locus coeruleus, resulting in altered sensitivity and gain regulation within insula-centered interoceptive networks. Such disruption of interoceptive processing may destabilize salience attribution, leading to impaired cognitive switching and attentional inefficiency, manifested clinically as brain fog.

EAT may transiently reshape these afferent inputs through mechanical stimulation of the nasopharynx while simultaneously engaging autonomic reflex pathways. Immediate effects may manifest as partial resynchronization of brainstem-level autonomic regulation, whereas continued EAT may gradually reduce chronic inflammatory signaling and restore the stability of interoceptive processing within the anterior insula and anterior cingulate cortex. Accordingly, the improvement in brain fog observed in this case is more coherently interpreted as reflecting neuroplastic recalibration of central regulatory networks rather than simple resolution of local inflammation.

Another noteworthy point is that recent studies have reported that EAT influences salivary amylase activity [18], which is regarded as an indicator of sympathetic nervous system activity centered on the locus coeruleus-noradrenergic (LC-NA) system. At the same time, involvement of non-LC-NA-mediated sympathetic pathways in salivary amylase responses has also been suggested, indicating that fluctuations in amylase activity do not simply represent sympathetic hyperactivation but may reflect qualitative alterations in stress response patterns.

In Long COVID, persistent stress load and heightened interoceptive sensitivity are thought to maintain excessive recruitment of arousal and attentional networks, including the LC-NA system, resulting in a neural state characterized by high arousal and reduced efficiency. Under such conditions, stimulus selection by the salience network (SN) may become overly sensitive, leading to excessive attribution of importance to minor internal sensations or external stimuli. This instability in attentional resource allocation is considered to worsen brain fog and fatigability.

Taken together, the improvement in brain fog observed in this case is best interpreted as an adjustment effect involving recalibration of central neural networks centered on the SN, rather than as a simple increase or decrease in sympathetic or parasympathetic activity. EAT may therefore restore cognitive clarity not by directly enhancing cognitive function, but by stabilizing neural decision-making processes that regulate interoceptive weighting and network switching. This perspective provides a hypothesis-generating framework that may help explain the potential effectiveness of EAT in conditions such as Long COVID and myalgic encephalomyelitis/chronic fatigue syndrome (ME/CFS), in which cognitive impairment occurs in the absence of overt structural abnormalities.

Thus, this case highlights the importance of understanding the effects of EAT not as a single mechanistic pathway, but as a staged, multi-layered process of biological regulation. The temporal dissociation observed between early improvement in autonomic-dependent peripheral manifestations, such as PVCs, and later improvement in central symptoms, such as brain fog, can be interpreted as reflecting reconstruction of multiple regulatory systems unfolding over different time scales. This clinical course supports the pathophysiological plausibility of both the EAT Reflex Adjustment Three-Phase Model and the EAT Field Theory when interpreted through the lens of temporal dynamics in symptom evolution.

This report represents a single-case observation, and therefore, causality between EAT and improvement in PVCs and brain fog cannot be established. In addition, the absence of objective assessments of autonomic and central nervous system function constitutes a limitation of this case. Future studies incorporating larger case series and objective autonomic and neurofunctional assessments are warranted.

## Conclusions

This report provides a novel clinical observation in which EAT was associated with improvement of both PVCs and brain fog in a patient with Long COVID, without pharmacological intervention. The temporal dissociation observed - earlier improvement in autonomic-sensitive peripheral PVCs followed by later improvement in brain fog, likely reflecting central autonomic network dysfunction - suggests that EAT may exert time-dependent and staged effects on autonomic regulation. Although causality cannot be established from a single case, these findings indicate that EAT may represent a non-pharmacological and minimally invasive therapeutic option for both peripheral and central manifestations of autonomic dysfunction in Long COVID.
